# Doctor at Your Fingertips: An Exploration of Digital Visits from Stakeholders’ Perspectives

**DOI:** 10.3390/life11010006

**Published:** 2020-12-24

**Authors:** Selina Demi, Samir Hilmy, Christina Keller

**Affiliations:** 1 Faculty of Computer Sciences, Østfold University College, 1757 Halden, Norway; 2 Mycronic, 183 53 Stockholm, Sweden; samir.hilmy@mycronic.com; 3 Department of Informatics, School of Economics and Management, Lund University, 220 07 Lund, Sweden; christina.keller@ics.lu.se

**Keywords:** digital visits, remote visits, telemedicine, remote real-time devices, telemedicine applications

## Abstract

Technological advances and the evolution of mobile technologies enable patients to meet their doctors through their smartphones. While offering the opportunity of digital visits to patients, there are also challenges for this development. The purpose of this study is to enhance the understanding of digital visits, as perceived by experts working in telemedicine companies. To serve this purpose, the authors conducted semi-structured interviews with managers and employees in eight telemedicine companies. The analysis of the empirical data confirmed the importance of digital visits and their efficiency. The potential of digital visits is expected to increase significantly, should they make use of remote devices to transfer real-time data from patients to physicians. In such a case, digital visits are expected to cover approximately 70–75% of medical cases. However, the use of remote devices must be taken with caution and specific conditions need to be taken into account. We encourage researchers to perform research on promising technologies such as artificial intelligence and remote diagnostic devices, which could make more diagnoses and conditions possible to be treated by digital visits. This is even more important in light of the ongoing Covid-19 pandemic.

## 1. Introduction

Healthcare has been considered the fastest growing industry in the world [1] and has tried for years to reap the benefits of the latest technologies to run in a more efficient manner. One way to achieve this is through telemedicine. Telemedicine can be defined as the use of electronic communications and information technologies to provide clinical services when participants are at different locations [2]. Throughout the course of history, people have attempted to share health information. Examples of this can be found in ancient civilizations where scrolls and hieroglyphs were used for that purpose [3] and during the fatal bubonic plague when bonfires were used as a means to exchange information across Europe [4]. Historians, however, believe that the first breakthrough in telemedicine can be traced to the invention of telecommunication devices [3]. In the 1900s, two-way communication became a possibility thanks to the invention of telephones, and physicians were amongst the first to use the technology. Radio communication was also a means of transferring medical information during World War I [4]. Decades later saw the introduction of the television, which added a whole new dimension to telemedicine. Video transmission for medical purposes was first used when the Nebraska Psychiatric Institute and the Norfolk State hospital communicated in 1964 [4]. However, it with the widespread use of mobile technology and wireless networks that telemedicine entered a new era in which common people had access to the technology and patients could finally make use of its benefits [3].

There are approximately 7 million patients using telehealth worldwide [5]. This number will only increase, as it is predicted that 75% of visits between patients and physicians could be carried out virtually [6]. Furthermore, advancements in mobile technologies during the late 2010s [3] have led to the development of telemedicine applications that aim to connect physicians and patients remotely [7]. In 2014, an explorative study was conducted with a goal to investigate patients’ experiences when communicating with physicians digitally [8]. Findings from interviews with 26 patients indicated accessibility as the main benefit of digital visits, given that patients do not need to travel, which is commonly associated with high costs, in particular for patients living in rural areas [8]. In the same year, the authors carried out focus group discussions with healthcare personnel from five primary healthcare centers in Sweden in order to explore their views with respect to digital visits [9]. Findings from these discussions indicate that digital visits enhance the collaboration between the physician and patient and make the physician more accessible. However, subjects of this study emphasized the need to evaluate costs and personnel resources when digitalizing medical visits. In the same line, previous research [10] supports the need to empirically evaluate the cost-effectiveness of digital visits. Moreover, it has been reported that the benefits of digital visits are theoretical and not yet validated [10]. With respect to costs, an evaluation study was conducted to compare the digital care model with the traditional in-office primary care in Sweden [11]. This study provided evidence that supports that the digital care model uses fewer resources than the traditional model and can lead to significant cost savings from the user and payer side.

In 2017, a survey was carried out with 399 virtual visits patients, followed by an observational study which took place in British Columbia, Canada [12]. As many as 93.2% of respondents were optimistic regarding digital visits and stated that digital visits provide high-quality care. Authors of the aforementioned study acknowledge the patient-centered healthcare enabled by means of digital visits, but also highlight the need to focus on its integration into existing healthcare systems. Recently, a qualitative study was conducted in order to explore viewpoints of 12 general practitioners on digital visits, by means of semi-structured interviews [13]. This study took place in two primary care centers located in North London. Despite the fact that respondents of this study confirmed the acknowledged benefits of digital visits, they also expressed concerns with regards to the usefulness of digital visits to patients and their ability to use technology, along with concerns regarding availability and quality of existing technological solutions. In addition, an extensive study on 35,000 patients in the time period of 2014–2017 demonstrated that digital visits reduced in-person visits by 33%, but at the same time increased total visits [14].

All this, along with the recent outbreak of Covid-19, which has led to close proximity to other human beings being discouraged, indicates that telemedicine is on the rise and telemedicine applications will have an even greater role to play in the near future. However, despite the promising potential, there is a limited number of studies that explore digital visits enabled by telemedicine applications from the perspective of stakeholders of telemedicine companies. Parimbelli et al. [15] organized two workshops with stakeholders of telemedicine systems such as developers, physicians, legal experts, and nurses and identified risks of two systems, namely, MobiGuide and AP@home. They identified risks such as data transmission problems, low quality of data, wrong data inserted by patients, failure to keep devices up-to-date, and harmful wrong indications, and concluded that telemedicine systems need to comply with existing regulations for broader adoption. Choi et al. [16] carried out a Delphi study with 190 telemedicine professionals including physicians, policy makers, patients, and industrialists. Their findings indicated physicians as the most opposed to the adoption of telemedicine. This reluctance was attributed to the lack of a delivery system for medical services and the possible disruption of primary care facilities. Sauers-Ford et al. [17] carried out a qualitative study by means of stakeholder interviews in order to understand why clinicians do not use telemedicine for pediatric patients. Their findings revealed benefits of telemedicine such as improving quality of care and decreasing costs, and challenges such as reluctance of physicians and technological challenges. Authors of this study acknowledge that the results might be biased since the stakeholders have pre-existing prejudices towards telemedicine. The majority of previous studies focus on understanding why telemedicine services are not widely adopted and their associated risks. On the contrary, the authors carried out a qualitative study with stakeholders of telemedicine companies in order to provide a holistic view of digital visits enabled by telemedicine applications.

Therefore, the purpose of this study is to enhance the understanding of digital visits, as perceived by stakeholders of telemedicine companies (An earlier version of this paper is available online [18]). To achieve this purpose, semi-structured interviews were conducted and thoroughly analyzed. Finally, the implications of this research are two-fold: to advance existing knowledge with respect to digital visits enabled by telemedicine applications and to encourage telemedicine practitioners to continuously innovate in order to augment digital visits. The findings reveal that emerging technologies such as artificial intelligence (AI) and remote diagnostic devices could enable digital visits to cover 70–75% of total consultations.

## 2. Materials and Methods

Despite the intrinsic potential of digital visits, they are not as widespread as traditional in-person visits. A plausible explanation of this could be the lack of knowledge regarding digital visits in terms of benefits, opportunities, and challenges. Therefore, to thoroughly explore the phenomenon of digital visits, this study adopts a qualitative approach [19], by means of semi-structured interviews [20] conducted during April–May 2019. A preliminary set of open-ended questions was used by researchers during the interviews, allowing for flexibility. The interview guide that was used during the interviewing process along with the codes used during the data analysis process are depicted in Table 1. 

The respondents were asked for permission to record interviews and their identities are not revealed throughout this paper. Nonetheless, their roles are disclosed, as the authors believe that such contextual information could add value and depth to the interpretation of empirical findings. The main requirement in selecting the respondents was that they were stakeholders working in telemedicine companies, as recommended in [21]. In order to gain a thorough and deep understanding of the opportunities and conditions to be fulfilled for these opportunities to take place, experts were chosen from different areas, as represented in the following table (Table 2).

It is noteworthy that more than half of the respondents’ roles are within the scope of management. Their perspective is important to understand not only the potential of digital visits, but also the constraints of these visits. The perspective of physicians was also considered given that they are the main users of new technological innovations in the field of telemedicine. Moreover, the investigation of new technologies in the context of digital visits dictates the need to tackle the business perspective, i.e., are these solutions feasible from an economic and technological perspective, are these technologies available or feasible to be developed? It is noteworthy that although patients are the core users of the service, they are not taken into consideration in this study. The authors state that this is outside the scope of the paper, given that the purpose is to explore digital visits from the standpoint of stakeholders working in telemedicine companies.

Interviews were recorded and transcribed by the first two authors, followed by an intense data analysis process. Initial realizations of the interviews were enhanced by posing follow-up questions when necessary. Given the explorative nature of this study, a vast amount of qualitative data that unveil stakeholders’ perceptions of digital visits was collected [19]. To systematically analyze the data, a thematic coding analysis technique was employed in order to extract themes from the data collected and to categorize them conceptually [22]. Themes and potential discrepancies were discussed among the three authors, and then further resolved.

## 3. Results

### 3.1. Digital Visits: An Overview

As reported by the respondents, the use of technology to support healthcare has a long tradition. It started on the internet with simple healthcare websites to support patients, and greatly diffused with the evolution of mobile technology. This is how one of the telemedicine companies that were explored in this study was founded:

*“When mobile technology took off, and people’s culture and attitude for devices changed, it became obvious there was an opportunity to bring health care to mobile devices. And that was the foundation for starting the company now nine years, which was that it ought to be possible to get your health care on your mobile device.”*

(Chief medical officer)

The paradigm of skepticism was initially reported by respondents, but interestingly, their skepticism was transformed after promising opportunities presented themselves over time.

*“So, I was a bit apprehensive as well as skeptical about it. But then, over a couple of years… I thought that this is probably the future of medicine. Because the research shows that 70–80% of the patients who are present to the general practice, they can be actually managed through video consultation, they don’t need to actually see the doctor face to face in a room. And they could be managed through video. So, I thought let’s give it a try.”*

In the attempt to understand the novelty that accompanies the concept of digital visits, experts agreed that real novelty does not lie in a new technology or service, but in a new model to distribute the same service. Respondents reported that digital visits were initially used for health guidance, redirection to appropriate healthcare, and providing prescriptions. However, with time, they expanded to accommodate physiotherapy consultations, midwife consultations, and follow-up chronic care. When it comes to diagnosis, respondents agreed that a relatively limited number of areas are currently covered. These areas are limited to flu, sore throat, tonsillitis, and skin conditions. Diagnoses that require a physical assessment such as ear infections or abdominal problems are not covered. Despite this limitation, respondents highlighted the wide patient target that could greatly benefit from the use of digital visits. For instance, students due to lack of time, senior citizens due to difficulties in going to the hospital in person, and patients with mental problems such as anxiety and depression *“…sitting in waiting rooms is a stigma attached to depression and anxiety…” (Physician)* or patients with chronic diseases *“…around 85% of the world’s health care costs is for chronic diseases… there is a lot of money involved…” (Business developer).* Interestingly, respondents identified tourists and truck drivers as potential users of digital visits who may benefit greatly.

### 3.2. Themes

The interviews were carefully analyzed by the authors and the following themes and supportive illustrations were retrieved (See Table 3). Empirical findings indicate evidence with regards to the benefits of digital visits, such as accessibility (theme 1), cost (theme 2), time (theme 3), quality of care (theme 4), and efficiency (theme 4). On the other hand, it was reported that digital visits face behavioral challenges (theme 1) and external/regulatory challenges (theme 2). Despite these challenges, experts believe digital visits show great potential, in particular, when making use of technologies such as artificial intelligence (AI) (theme 1) and remote diagnostic devices to transmit real-time patient data to doctors (theme 2). Conditions that need to be considered along with the aforementioned opportunities are represented as the following guidelines: integration (theme 1), distribution and logistics of remote devices (theme 2), cost of remote devices (theme 3), and device testing (theme 4).

We summarize the main findings in the following table (Table 4):

## 4. Discussion

In this section, the authors interpret their empirical findings in relation to existing literature, where available. Firstly, an overview of digital visits consisting of initial perceptions of respondents, types of services, and types of patients who may benefit is presented. This section is followed by the discussion regarding the themes identified, i.e., benefits, challenges, future opportunities, and guidelines. Moreover, limitations of this study are presented and finally, implications for practice and research are identified.

### 4.1. Digital Visits: An Overview

Findings of this study indicate initial concerns with regards to digital visits among respondents, in particular, physicians. A certain skepticism from physicians has been reported in literature over years [13,23]. One may explain this by the fact that the digitalization of the service might cause a disconnection between patients and physicians, which in turn could lead to bad medical practices. Contrary to physicians, business experts have, since the beginning, believed in digital visits as the future of healthcare. One explanation of this discrepancy could be related to the area of interest of respondents, i.e., one group focuses on the way of working while the other perceives it as a business opportunity. 

According to one of the respondents, digital visits are able to cover only 30–35% of total consultations. Hence, limitations do exist, and they are related to the difficulty in diagnosing all symptoms digitally. This finding is also acknowledged by previous research in the field [9,24]. Respondents revealed examples of areas that require human touching or lab tests, and therefore cannot be treated digitally, as supported by previous research [13]. With respect to this acknowledged limitation, authors of this study feel optimistic by perceiving it as appealing for further research. Furthermore, the findings support that patients with chronic diseases could benefit the most from the use of digital visits. Additionally, this study identified other potential categories such as students [12] and senior citizens [10,24]. Regarding patients with mental issues, one of the respondents highlighted that they could benefit from digital visits to avoid sitting in waiting rooms, which is a stigma attached to depression and anxiety. In this regard, controversial views are observed in [13], with some respondents perceiving these patients as potential users of digital visits, and others highlighting the difficulty with which patients and physicians communicate in remote settings. There was also evidence that patient categories such as tourists and truck drivers could benefit from digital visits, as they are not in the same geographical area as their physical healthcare center. 

### 4.2. Benefits

This study provides evidence about accessibility being the main benefit of digital visits. Patients who live in rural areas, those with disabilities, and the elderly are able to access healthcare digitally due to its convenience, as supported in the literature [8,10]. However, a discrepancy was noted with respect to the elderly. While it is acknowledged that digital visits are more convenient for the latter, respondents also indicate that age and accessibility are inversely proportional due to the elderly having difficulties with the use of technology. Furthermore, digital visits bring cost savings [10,12] from two perspectives, namely, patients and the healthcare system. Regarding the former, it was reported that the cost of a digital visit is three times less than a physical consultation, along with other savings such as travel expenses or opportunity costs. Additionally, digital visits are reported to perform with twice the efficiency of physical visits. As for the healthcare system, digital visits use fewer resources, e.g., physicians’ offices and urgent care, therefore, they save costs. A study conducted in 2017 in Sweden confirmed that costs of digital visits are less than in-person visits [11]. However, considering the limited adoption of digital visits, these savings were not yet significant for the healthcare system.

Furthermore, previous studies support the time-effectiveness of digital visits in terms of waiting time and consultation time. Two out of nine respondents revealed that patients have to wait approximately 10 min once they send their request, which is significantly lower compared to in-person visits where patients may wait up to two weeks. With respect to consultation time, two out of nine respondents reported a lower consultation time during digital visits compared to in-person visits. The authors of this study interpret this finding due to the fact that during digital visits, it is possible for physicians to have patient information beforehand and this decreases consultation time. On the contrary, two out of nine respondents did not support this finding and explain it by two factors, i.e., flexibility and accessibility. One plausible interpretation of this could be that digital visits are flexible in allowing visits of more than 10 min without extra charges, and the high accessibility introduces the risk of overusing the system. 

Additionally, with respect to quality of care, one may expect a lower quality in the case of digital visits. In fact, one of the respondents presented an interesting argument in support of digital visits. According to this respondent, automation and reliance on computers enhances accuracy and adherence to guidelines. In these regards, one of the telemedicine companies was reviewed for urinary tract infection among women and achieved 99.6% compliance to national guidelines compared to approximately 70% compliance of primary care. However, the aforementioned finding is the only evidence in support of quality of care as a benefit of digital visits, therefore this finding is not conclusive. Overall, it was observed that quality of care is more dependent on the physicians’ experience and capability, rather than the setting of the visit. 

### 4.3. Challenges

The respondents reported that the bond created between patients and physicians is stronger during in-person settings than digital visits. This has also been reported in literature [25]. The reluctance of physicians towards digital visits can be attributed to patient empowerment which has threatened the authoritative power that physicians traditionally have over patients [26]. Additionally, the respondents mentioned the reluctance of patients, elderly patients in particular, towards digital visits and attributed this reluctance to their difficulty with using new technologies.

Moreover, it was noted that respondents were mostly concerned about external challenges. The majority of respondents who worked in telemedicine companies based in the US (56%) reported that physicians need to be licensed in each state they intend to practice, as a relevant barrier towards the adoption of digital visits. In Sweden, regulations depend on the region and this was identified as problematic by one of the respondents. Moreover, three out of nine respondents highlighted reimbursement as another challenge, with two of them suggesting improvements in existing reimbursement models. Given that physicians are not able to see patients, there is a risk for inaccurate diagnosis; therefore, a large indemnity demanded by physicians is not surprising. Physicians have traditionally demonstrated reluctance to adopt new technologies unless they were reimbursed, as was the case with the introduction of electronic health records [26].

### 4.4. Future Opportunities

Findings revealed the potential of digital visits to augment and cover a larger number of medical cases:

*“…what we’ll see over time is a move to a large proportion of cases, visits happening virtually, as I say, if it’s possible for a visit to be virtual, why on earth would you want to do it in person?”*

(Chief medical officer)

This potential may be realized by means of new technologies such as AI. In Sweden, for instance, customized treatment based on patient data is a crucial part of the national e-health vision for 2025 [27]. The usefulness of using AI from the viewpoint of telemedicine companies lies in the collection of patient data which, in turn, enables them to better understand patients’ needs and to make accurate decisions. In addition to the datafication of patients, an interesting future opportunity lies in intelligent virtual caregivers that will monitor patients. Although the telemedicine companies under study had started using this technology to some extent, an implicit hesitation was observed. One plausible explanation may be the risk of dehumanization, identified also as part of the 4D (depersonalized, discriminatory, dehumanized, disciplining care) risk model of AI in elderly care [28]. This means that the use of virtual caregivers and the datafication of patients may lead to the loss of human interaction.

Use cases of digital visits are limited given that in specific circumstances, diagnostic devices may be needed. Interestingly, one of the respondents presented the idea of putting devices that are needed for diagnosis into the hands of patients: *“the notion that a symptom cannot be diagnosed because you need a physical component is not actually true. Because what you can do is that you can put the physical device that you need to use for diagnosis in the hands of the patient…” (Chief business development officer).* Ideas about remote devices that transmit data in real-time from patients to doctors were introduced by respondents such as the spirometer for patients with asthma to monitor their oxygen uptake or a device to listen to the heart and lungs of the patient. Few of these devices are on trial and may be used in a few years, however, data about these devices was not provided due to confidentiality constraints. Should these medical-grade devices be used by patients and transmit data in real-time to physicians, e.g., to measure oxygen uptake and to listen to the heart and lungs, digital visits are expected to experience significant augmentation. One of the respondents revealed that without these devices, 30–35% of total consultation can be done digitally, but with the incorporation of these devices, approximately 70–75% of consultations could be done digitally. Therefore, we deduce a promising expansion of digital visits, acknowledging that its adoption will take time. According to the diffusion of innovation theory, the adoption of a new idea does not occur simultaneously in a social setting, but it is rather a process where some participants are more predisposed to adopt it than others [29]. 

### 4.5. Guidelines

The respondents provided the authors with useful insights into the requirements that need to be considered when planning to incorporate remote devices for diagnosis with digital visits. Distribution and logistics of these devices was one of the aspects identified, in terms of how to fix the settings of these devices, how to distribute them, how to support users in case of problems, and how to connect these devices with physicians. As can be noticed, these issues weigh mostly on the patients’ side. Therefore, training patients for the use of such devices will be necessary. Additionally, the existing high cost of medical devices may lead to inaccessibility of medical care for a considerable part of the society. The respondents also highlighted the need to test the quality and accuracy of the devices that will be used by patients and transmit data in real-time to physicians. This is not surprising because inaccuracies could have serious consequences on patients’ health. Therefore, guidelines on how to make sure these devices work properly, i.e., are measuring what they are intended to measure in a correct manner, must be in place. Finally, data from these diagnostic devices and digital visits platforms in general need to be integrated and coordinated with general care.

### 4.6. Limitations of the Study

The main limitation of this study is the low number of respondents. One explanation of this is the limited number of telemedicine companies and practitioners working in these companies. Considering this fact, generalizations of findings to other contexts must be made with caution. To mitigate this limitation, the sample of subjects of this study covered a diversity of telemedicine companies and experts from a variety of disciplines, e.g., business, technology, management, physicians. Therefore, we believe that although the number of respondents is low, the inclusion of multiple cases allows us to derive conclusions that are generalizable, to some extent, to other telemedicine companies. It is noteworthy that the perception of patients was not captured in this study, as authors focused on experts’ viewpoints on the matter [21].

Due to the explorative nature of this study, ensuring the reliability of the data analysis process is not trivial. In this regard, empirical data retrieved by semi-structured interviews were analyzed by means of thematic coding technique. Authors designed a preliminary coding scheme which was used and evolved throughout the analysis process. The coding process was conducted by the first author and cross-checked by the second author, to avoid potential mistakes that could lead to erroneous interpretation of findings. To ensure the traceability of data collection, analysis and conclusion, authors provide an archived replication package [30].

### 4.7. Implications for Practice and Research

According to the findings of this study, we encourage researchers to focus on the following dimensions in order to fully reap the benefits of digital visits:

- Measure the quality of digital visits by considering patient-centered outcomes and compare them with in-person visits. With regard to this, Kahn [10] suggested metrics such as mortality and functional status. Other metrics could also be used such as the number of digital visits redirected to in-person healthcare and the number of digital visits in which physicians were able to provide correct diagnoses.
- Investigate how chronic patients may benefit from the use of digital visits, given that this patient group was identified by respondents as very promising.
- Explore in detail the future opportunities of digital visits, with a particular focus on remote real-time devices for diagnosis and monitoring. Identify best practices and guidelines for the use of these devices in support to digital visits, considering aspects such as integration, distribution and logistics, cost, and device testing.
- Explore the potential of using digital visits in the context of a pandemic such as COVID-19, starting small with health guidance and direction to the right healthcare. In fact, a recent systematic review demonstrated the significance of using digital care during the Covid-19 pandemic [31]. Ambitiously, this idea may evolve into embracing the concept of remote testing and transmission of patient data to doctors in real-time. The authors expect this to take time and effort, but it seems to have promising effects such as reducing the strain on resources of medical centers during a pandemic and reducing the spread of infection by minimizing exposure.

Moreover, telemedicine companies need to continuously innovate in order to augment digital visits. Stakeholders should focus on the use of promising technologies such as AI and, in particular, remote devices for diagnosis. They must strive for the integration of these remote devices with their existing services and plan carefully by considering aspects such as distribution and logistics, cost, and device testing.

## 5. Conclusions

Digital visits are on the rise and they are expected to play an important role during the ongoing Covid-19 pandemic. The authors of this study intend to enhance the knowledge regarding digital visits by conducting semi-structured interviews with stakeholders of eight telemedicine companies. The results of this study identified the following groups that could benefit from meeting physicians through their smartphones: patients with chronic diseases, patients with mental issues, students, senior citizens, tourists, and truck drivers. The findings also indicate the main benefits of digital visits: accessibility, cost-effectiveness, time-effectiveness, and quality of care. While respondents outlined digital visits as twice more efficient than in-person visits, they acknowledged behavioral challenges in terms of reluctance of physicians in adopting digital visits and difficulties of elderly patients in using new technology, as well as external challenges related to regulations.

According to the respondents, digital visits currently cover approximately 30–35% of total consultations; however, enabling patients to use smart diagnostic devices that transmit data to physicians in real-time has the potential to augment digital visits. In such a case, approximately 70–75% of the total consultations can be done digitally. We invite researchers to develop guidelines and best practices related to the logistics, distribution, cost, and testing of these smart devices.

Our future work will focus on smart diagnostic devices that transmit data in real-time from patients to physicians. Empowering patients by putting these devices into their hands is an ambitious endeavor but can be valuable, particularly during a pandemic.

## Figures and Tables

**Table 1:** Interview guide, themes, and codes.

Themes	Codes	Illustrative Question
Benefits	Ben	What in your opinion are the benefits of digital visits?
Accessibility Cost Time Quality of care	Ben–Acc Ben–Cost Ben–Time Ben–Qua	Accessibility Cost Time Quality of care
Challenges	Chall	What in your opinion are the challenges of digital visits?
Behavioral External/Regulations	Chall–Beh Chall–Ext	Behavioral External/Regulations
Future Opportunities	FOpp	What needs to be done in the future to augment digital visits?
Customization Remote real-time devices	FOpp–Cust FOpp–Rrd	
Guidelines	Guide	What conditions need to be in place to make use of remote diagnostic tools to augment digital visits?

**Table 2:** Respondents’ roles.

Roles of Respondents	Number of Interviews
Physicians	2
Business developers	2
Chief Medical Officer	2
Chief Business Development Officer	1
Head of Innovation	1
Chief Executive Officer	1
Total	9

**Table 3:** Themes and illustrations.

Theme	Illustration
Benefits theme 1 Accessibility	“*Elderly people are quite happy to use professional collaboration, which is very easy to use, from the patient’s perspective, because I see so many elderly people who just log in themselves*…” (physician)
Benefits theme 2 Cost	“*When you go to a doctor and on average a doctor’s visit is about $30, you are going to pay the entire amount, until you reach your deductible. In our case, the price can be for as little as $5 to $10 a month…”* (Chief executive officer) “*The healthcare sector saves money because they reimburse the physician. Healthcare system already spends a lot of money in hospitals, urgent care or in physicians’ offices, because they know the hospitals supports so many employees and the surgeon center supports many. So, the less the physician is spending the money, you will be willing to take this money and the health care will be better*…” (Physician)
Benefits theme 3 Time	“*If I, and even as a doctor myself, know I am ill and want to know what is wrong with me and what kind of medication I need, I would rather go to a digital doctor, rather than making an appointment with my GP and wait for 2 weeks to be seen*…” (Physician)
Benefits theme 4 Quality of care	“*We were reviewed a couple of months ago, and for a very large patient group, which is a urinary tract infection among women. And we had 99.6% of adherence to national guidelines, which is basically unheard of the usual number is somewhere around 70% in standard healthcare. And the reason why we can be so much better is because we can automate basically everything. And basically, machines never, never forget to ask questions or never do not follow guidelines*…” (Chief business development manager)
Benefits theme 5 Efficiency	“*The typical Min Doktor can handle between 15 and 20 patients an hour. And in the physical setting, if you are a really fast doctor, I think you can perhaps handle 7–8 at the maximum. So, it’s probably twice the efficiency*…” (Head of innovation)
Challenges theme 1 Behavioral	“*Now it’s more of an equal collaboration with a doctor and patient, and of course, some doctors are really intimidated or frightened by this change*…” (Head of innovation)
Challenges theme 2 External	“*I think with time they will be overcome because of the quality and positive impression from the people in that field will prevail and the movement will be accepted and the laws will be in its favor*…” (Physician)
Future Opportunities theme 1 Customization	“…*artificially intelligent virtual caregiver that is going to revolutionize the whole of healthcare*…” (Chief executive officer) “*I don’t know because this technology does not feel the emotions or the stress of the patient*…” (Physician)
Future Opportunities theme 2 Remote real-time devices	“*There is the potential to care for approximately 70 to 75% virtually if you combine physical components from the perspective of the patient*…” (Chief business development manager) “*We are piloting seven different devices focusing on one, a device with which you can listen to the heart and listen to the lungs, look into the ears and into the throat of patients*…” (Chief business development officer)
Guidelines theme 1 Integration	“*We need a better national or nationwide infrastructure. It’s quite fragmented at the moment, due to that we have a regional focus on technical solutions, for example, bigger nationwide database*…” (Head of innovation)
Guidelines theme 2 Logistics	“*Logistics in the sense that you have to fix the setup for how do you as a patient get hold of, for instance, a glucose meter? How is that being distributed? Who is doing the service and support when things are not working well? How do you connect that medical device to someone basically, who answers on the other side of the phone?*” (Chief business development officer)
Guidelines theme 3 Cost of devices	“*It’s all about the price. If Apple introduces some external device, which is like for hundreds and hundreds of pounds, people will not use it. But when it becomes automated, and they are available for 10 pounds and 15 pounds, then everybody will use. So, it all depends on availability. So, I’m looking at maybe in the next couple of years, more and more people will be using these external devices*…” (Physician)
Guidelines theme 4 Device testing	“*You need to have verified measurements tools, like whatever these diagnostic real time devices intake, when you taking diagnostic areas, whatever its oxygen saturation, or heart rate, or whatever, they need to be CE qualified, they need to be approved*…” (Business developer)

**Table 4:** Findings.

Themes	Findings
Benefits	Digital visits are more convenient for patients living in rural areas, patients with disabilities, and elderly patients. Lower costs for patients (three times cheaper than an in-person visit) and cost-effective from the healthcare system perspective (twice more efficient than in-person visits) Lower consultation time and lower waiting time (10 min) Automation enhances accuracy, and therefore the quality of care (inconclusive finding).
Challenges	Reluctance of physicians and patients (elderly) towards digital visits Loss of authoritative power of physicians and empowerment of patients State-based or region-based regulations The need to revise reimbursement models
Future Opportunities	Customized treatment using patient data and AI technology Virtual caregivers to monitor patients Diagnostic devices that transmit data from patients to doctors in real-time The use of these diagnostic devices could enable 70–75% of total consultations to be done digitally
Guidelines	The respondents outline the need to plan on how to distribute diagnostic devices, how to connect them with physicians and how to support patients in using them. The respondents recommend a lower cost of these remote devices to make them more accessible. The respondents outline the need to test these devices in order to evaluate that they are measuring and what they are intended to measure in an accurate manner. The respondents suggest coordinating and integrating data from these remote devices with the general healthcare system.

## Data Availability

The data presented in this study are openly available in FigShare at [10.6084/m9.figshare.12950123], reference number [30].
